# Synthesis and characterization of 3D topological insulators: a case TlBi(S_1−*x*_Se_*x*_)_2_

**DOI:** 10.1088/1468-6996/16/1/014405

**Published:** 2015-02-25

**Authors:** Kouji Segawa

**Affiliations:** Institute of Scientific and Industrial Research, Osaka University 8-1 Mihogaoka, Ibaraki, Osaka 567-0047, Japan

**Keywords:** topological insulator, Dirac semimetal, topological phase transition

## Abstract

In this article, practical methods for synthesizing Tl-based ternary III-V-VI_2_ chalcogenide TlBi(S
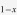
Se_*x*_)_2_ are described in detail, along with characterization by x-ray diffraction and charge transport properties. The TlBi(S
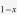
Se_*x*_)_2_ system is interesting because it shows a topological phase transition, where a topologically nontrivial phase changes to a trivial phase without changing the crystal structure qualitatively. In addition, Dirac semimetals whose bulk band structure shows a Dirac-like dispersion are considered to exist near the topological phase transition. The technique shown here is also generally applicable for other chalcogenide topological insulators, and will be useful for studying topological insulators and related materials.

## 1. Introduction: Tl-based topological insulators

Topological insulators have attracted great interest because of their peculiar surface state, which hosts spin-polarized Dirac electrons [1–3]. Possible motivations for studying topological insulators are utilization of the surface state, such as transparent electrodes [4], and/or exploring exotic phenomena. To date, one of the necessary conditions for a material to be a topological insulator is occurrence of band inversion due to strong spin-orbit interaction, and therefore topological insulators must contain heavy elements like Bi. The prototypical topological insulator is tetradymite Bi_2_Se_3_ [5, 6], which many three-dimensional (3D) topological insulators are rooted on. Bi_2_Se_3_ has a so-called quintuple-layer structure, which consists of Se–Bi–Se–Bi–Se layers coupled to one another with a van der Waals gap. In many 3D topological insulators like PbBi_2_Te_4_, GeBi_2_Te_4_, GeBi_4_Te_7_, etc [7–12], the unit structure is a similar kind of multiple layer; for example, septuple layers of Te–Bi–Te–Ge–Te–Bi–Te are formed in GeBi_2_Te_4_. The symmetry of the crystal structure is rhombohedral, and it is essentially unchanged from Bi_2_Se_3_. The unit structure of Bi_2_Se_3_ tetradymite is understood as a distorted rock-salt-like structure that is elongated along the (111) direction, and many brother systems take similar structures. Reduction of symmetry from cubic to rhombohedral is likely to be necessary for band inversion at odd numbers of time-reversal symmetry momenta, which causes nontriviality [13].

To date, there are not many variations of the crystallographic systems of 3D topological insulators characterized by a *Z*_2_ invariant (*Z*_2_ topological insulators), although more than 20 systems have been discovered as *Z*_2_ topological insulators [3]. However, there is a very different and intriguing system among them: a series of Tl-based ternary topological insulators such as TlBiSe_2_. There are many interesting features to these materials. (1) They have a topologically *trivial* compound whose crystallographic structure is identical and continuously connected to nontrivial phase. In other words, the topological phase transition is realized in these systems, which is rarely seen in *Z*_2_-topological-insulator systems. (2) A 3D Dirac semimetal is expected to be realized, in relation with the topological phase transition [14, 15]. (3) There is a system in which the surface Dirac cone has a gap, although there is no factor which breaks the time-reversal symmetry [16]. (4) In particular for TlBiSe_2_, the band structure of the surface state is quite simple and the Dirac cone is placed at the 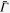 point. (5) The bulk band gap is ∼0.35 eV, which is the largest among known topological insulators and important for application at high temperatures. (6) No van der Waals gap takes place in their structure. This can be a demerit since scanning tunneling microscope/spectroscopy cannot be applied to these systems.

The first time that the systems attracted interest as topological insulators was in February 2010. Two reports of the theoretical prediction of the realization of the topologically nontrivial phase in Tl-based ternary III–V–VI_2_ compounds were posted independently [17, 18]. At first, TlBiTe_2_ had been considered to show superconductivity at low temperatures [19], but now people realize that the superconductivity is due to an impurity in TlTe [20]. Although superconductivity has nothing to do with this, interest in a possible new topological insulator system remained. The first report of an observation of a Dirac cone in TlBiSe_2_ was posted in June 2010 by Sato *et al* [21], and it was followed by two independent groups [22, 23].

The nontriviality of TlBiSe_2_ and TlBiTe_2_ was certainly predicted [17, 18]; however, reality turned out to be different from the prediction on TlBiS_2_, as the real structure is completely different from the basis of the prediction in TlSbS_2_ and TlSbSe_2_. Experimentally TlBiS_2_ is revealed to be topologically *trivial* by angle-resolved photoemission spectroscopy (ARPES), and this fact has been reported by two groups independently [16, 24]. The structure of the trivial TlBiS_2_ is qualitatively identical to that of the nontrivial TlBiSe_2_, and thus it is obvious that there is some point at which a phase transition from trivial to nontrivial takes place. Indeed, the topological phase transition is observed [16, 24], and the transition point turns out to be close to *x* = 0.5 of TlBi(S
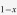
Se_*x*_)_2_ [24]. This feature gives us significant help in determining whether some phenomenon originates from the topological surface state. If such a phenomenon is not observed in TlBiS_2_ but observed in TlBiSe_2_, we can easily say that has a topological origin. Therefore, the system TlBi(S
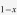
Se_*x*_)_2_ provides a useful platform to study the topological surface state experimentally. Furthermore, between the trivial and nontrivial phases, a Dirac semimetal whose 3D band structure has a Dirac-cone dispersion is considered to realize [25, 26].

Another striking feature is the Dirac gap in TlBi(S
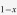
Se_*x*_)_2_ for 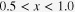. Sato *et al* reported that a gap opens in the surface Dirac cone if the composition is changed from TlBiSe_2_ toward TlBiS_2_ while keeping the composition within that of the nontrivial phases. This feature is quite unusual because breaking the time reversal symmetry is necessary to open the gap in the Dirac cone. One may suspect that the gap is due to hybridization of some imperfect cleavage of the sample, but this is not likely because the gap opening is very reproducible in experiments by Sato *et al* [16]. The origin of the gap has been proposed by theorists [27], but it is still puzzling.

In this paper, I describe the synthesis and characterization of the intriguing system, TlBi(S
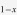
Se_*x*_)_2_, which is useful for studying the topological phase transition, Dirac semimetals, and the mechanism of the unexpected opening of the gap in the topological surface-state dispersion.

## Single crystals of TlBi(S
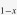
Se_*x*_)_2_

2.

### 2.1. Structure

**Figure 1.**
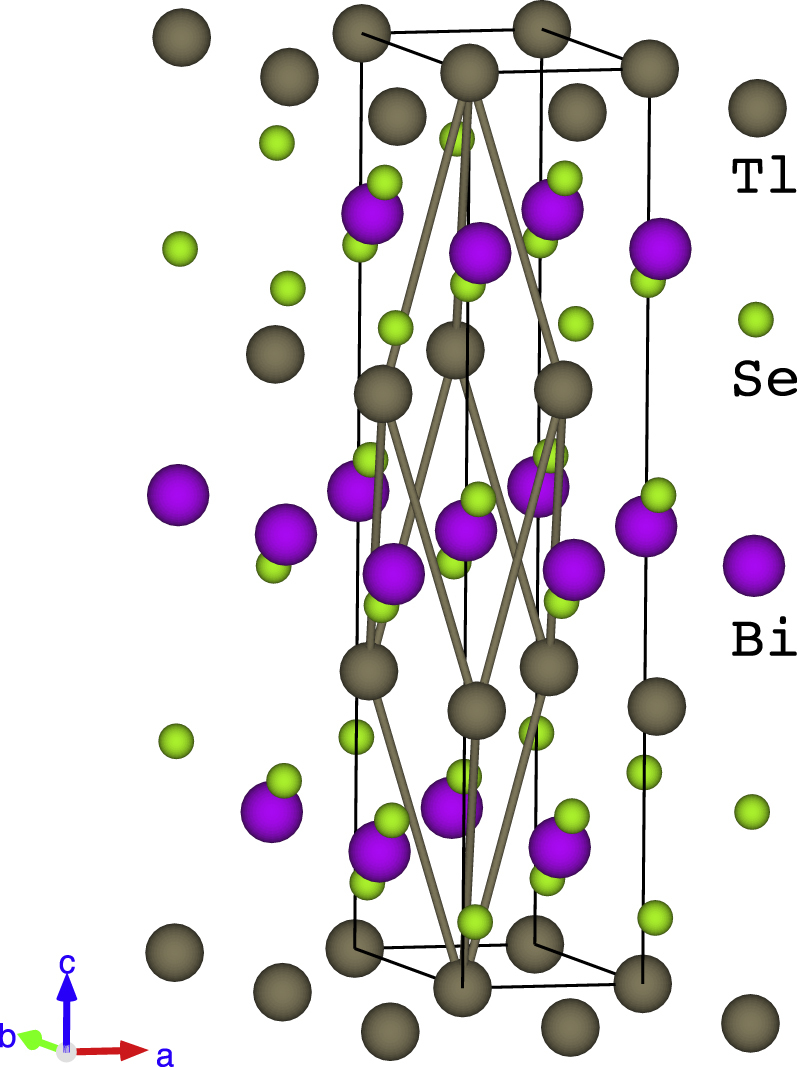
Crystal structure of TlBiSe_2_. Hexagonal unit cell is shown by black thin lines, and the rhombohedral unit cell is shown by gray thick lines [34].

Figure 1 shows the structure of TlBiSe_2_, which is called an NaFeO_2_-type. The crystallographic structure was first reported for TlBiTe_2_ in 1961 [28], and the structures of TlBiSe_2_ and TlBiS_2_ are essentially identical. The space group is R 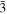 m (No. 166), which is the same as for other *Z*_2_ topological insulators such as Bi
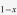
Sb_*x*_, Bi_2_Se_3_, and tetradymite families such as GeBi_2_Te_4_, etc. Bi
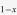
Sb_*x*_ takes a face-centered cubic (fcc)-like lattice structure distorted along the (111) direction. The unit structure of tetradymite is similar to that of TlBiSe_2_ and TlBiS_2_, but it continues for only five layers like Se-Bi-Se-Bi-Se because of the charge neutrality. In the TlBiSe_2_ and TlBiS_2_, Tl^+^ and Bi^3+^ align alternately, the averaged valence of the cations is +2, and charge neutrality is naturally conserved. Therefore, the structure of TlBiSe_2_ and TlBiS_2_ can be understood as a distorted NaCl-type, in which Tl^+^ and Bi^3+^ are alternately placed at the cation sites. The lattice constants were given in multiple papers [30–33], and the results are essentially consistent. Please note that the number of independent parameters is two, and thus the parameters of the hexagonal unit cell can be converted into a rhombohedral unit-cell expression, and vice versa. The conversion formulae are shown in the appendix.

### 2.2. Synthesis

Single crystals of most chalcogenide topological insulators are made by a melt-grown method. The Bridgman method is one kind of melt-grown method. Since the compound melts congruently, the growth process consists of slowly cooling down a melt under a certain temperature gradient. The samples must be sealed in a quartz tube, because the vapor pressure of chalcogenides is usually high at high temperatures. For TlBiSe_2_ and TlBiS_2_ the melting temperature is low, and the reaction between the melt and a quartz tube is negligible. Synthesis of those systems has already been reported [30–33]. A ternary phase diagram was also investigated for both TlBiSe_2_ and TlBiS_2_ [35–37], and it is known that these compounds melt congruently. The growth method is essentially the same in all reports, and thus I introduce our method for synthesizing crystals as an example.

**Figure 2.**
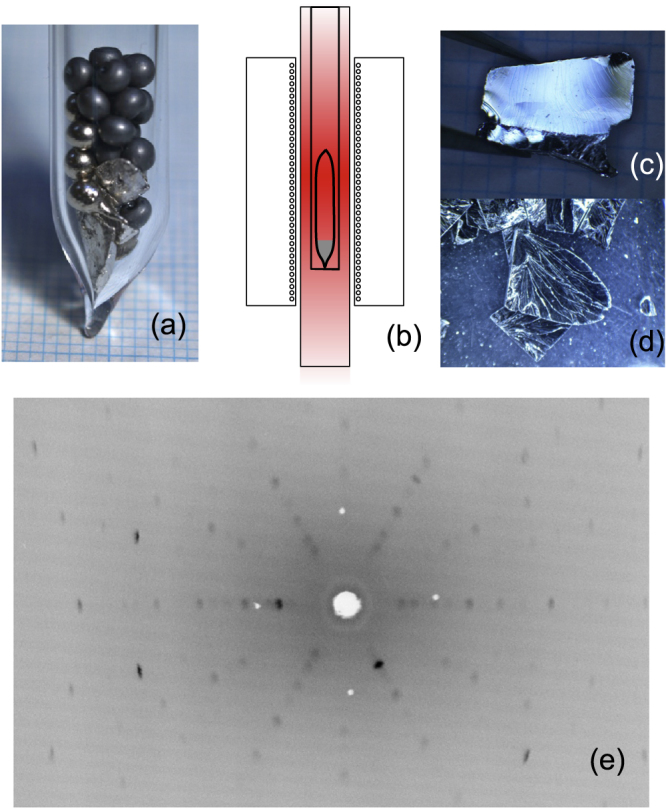
(a) Raw materials put in a sealed quartz tube. Round black shots are selenium, round silvery shots are bismuth, and irregularly shaped pieces are thallium. The outer and inner diameters of the quartz tube are 9 mm and 7 mm, respectively. (b) Schematic of the growth, where the sealed quartz tube is put at a slightly lower position in the furnace. (c), (d) Pictures of cleaved crystals of TlBiSe_2_. (e) Laue diffraction pattern of TlBiSe_2_ on the cleaved surface.

A picture of the starting materials sealed in a quartz tube is shown in figure 2(a). The tip of the quartz tube is narrowed to restrict nucleation of the growth at the beginning. This is useful for obtaining crystals of larger domains. The purity of starting materials of Tl, Bi, Se, and S is 99.999%(5N), 99.9999%(6N), 5N, and 5N, respectively. To remove the oxidized surface of the raw materials, Tl shots are annealed in a hydrogen atmosphere at 270–300 °C and Bi shots are washed with a diluted HNO_3_ solution. The amount of the raw materials is carefully controlled in the glove box, and the materials are mixed into the quartz-glass tube, which is sealed after evacuating with a diffusion pump and then filled with a small amount of pure argon. The ratio of the materials is stoichiometric in the present experiment, but recently, the shifted composition of starting materials turned out to be useful for synthesizing bulk-insulating samples [38, 39]. Before the main growth procedure, the raw materials are slowly warmed up at the rate of 100 °C/h and kept at 900 °C to complete the reaction. At 900 °C, the quartz tube is shaken to homogenize the melt and eliminate bubbles.

A schematic picture of the growth setup is shown in figure 2(b). In the present experiment, I used a vertical tube furnace with a single temperature control. A natural temperature gradient is used for growth, and it is measured with thermocouples to be ∼10 °C/cm. The vacant part of the quartz tube is placed at a higher temperature position in the furnace to prevent condensation of vapor into any other unexpected phase. For growing TlBiSe_2_, the temperature-sweep rates of 0.5, 1, and 2 °C/h are tried, and the best value among them is determined to be 1 °C/h by evaluating the transport properties (details are shown below). As for the solid-solution compounds, the temperature sweep rate of 2 °C*/*h is applied to grow TlBi(S
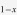
Se_*x*_)_2_ crystals, including TlBiS_2_, because comparable quality is achieved in the growth of TlBiSe_2_ at the rate of 2 °C*/*h. The melting points of TlBiSe_2_ and TlBiS_2_ are slightly different: 720 °C [40] and 740 °C [32], respectively. Therefore, the growth conditions of TlBi(S
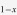
Se_*x*_)_2_ are changed for *x*: The temperature sweep range for solid-solution compositions is from 860 °C to 660 °C for *x* 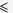 0.3, and from 840 °C to 640 °C for *x* 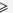 0.4.

## 3. Characterization: x-ray diffraction

**Figure 3.**
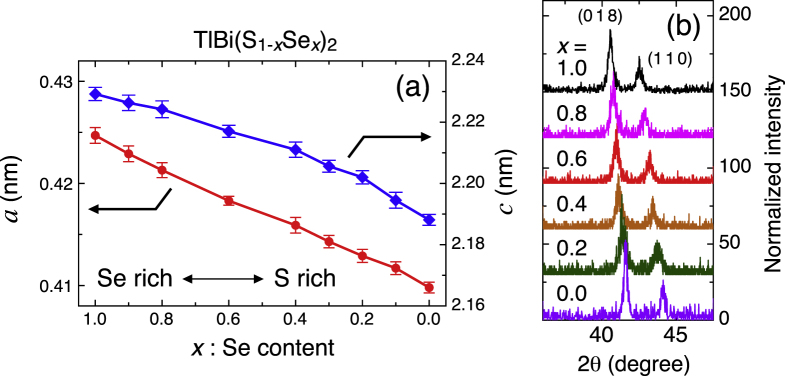
(a) Lattice constants determined from power XRD are plotted as functions of the Se content. (b) Powder XRD patterns. Normalized intensity profiles of the (018) and (110) peaks in the powdered TlBi(S
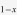
Se_*x*_)_2_ single crystals for a series of Se concentrations; the (018) peak reflects both the in-plane and out-of-plane periodicities, whereas the (110) peak reflects solely the in-plane periodicity. Taken from [16].

Figures 2(c) and (d) show photographs of TlBiSe_2_ crystals. The obtained crystals are easily cleavable, and the grain size is as long as ∼5 mm along the cleavage plane. Figure 2(e) shows the Laue picture taken with respect to the cleavage plane. This clearly shows the three-fold symmetry of the system, and the result is consistent with the structure of TlBiSe_2_. The lattice constants of TlBi(S
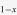
Se_*x*_)_2_ are obtained by both single-crystal and powder x-ray diffraction (XRD), as shown in figure 3. If I take a hexagonal lattice, 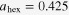
–0.410 nm and 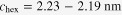 for *x* = 1.0 to 0.0. It is important to note that the lattice parameters change *continuously* with changing *x*, and the crystallographic structure does not change.

**Figure 4.**
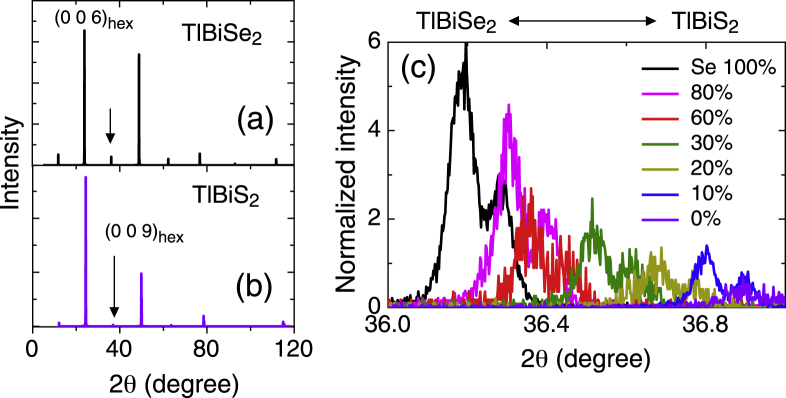
(a, b) Single-crystal XRD patterns of TlBiSe_2_ and TlBiS_2_ taken on the *c*-plane. Arrows show peaks at (0 0 9)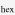. (c) Profiles of TlBi(S
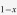
Se_*x*_)_2_ around the (0 0 9)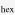 peak normalized by the intensity of (0 0 6)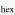 peaks. The normalized intensity shows a clear decrease with decreasing *x*.

Figures 4(a) and (b) show the single-crystal x-ray profile of TlBiSe_2_ and TlBiS_2_. Apparently the intensity of 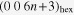 peaks with integer *n* is much weaker in TlBiS_2_. Figure 4(c) shows (0 0 9)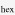 peaks normalized by those of (0 0 6)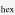 for various compositions of TlBi(S
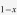
Se_*x*_)_2_. The intensity decreases with a change in the composition from TlBiSe_2_ to TlBiS_2_. As described above, the crystal structure of a series of the Tl-based ternary compound can be understood as an fcc structure distorted along the (111) direction [17, 29]. However, the displacement of chalcogen sites from the fcc position and the alternate order of Tl and Bi causes longer periodicity and produces (0 0 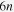
+3)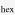 peaks in the XRD profile. Therefore, the smaller intensity of (0 0 6*n*+3)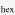 peaks suggests that the displacement of chalcogens in TlBiS_2_ is smaller than that in TlBiSe_2_. In the calculation by Lin *et al*, the displacement of chalcogens is a key for producing band inversion [17]; this may be an origin of the absence of band inversion in TlBiS_2_, along with weakness of the spin-orbit interaction due to the lighter element, S.

## 4. Characterization: transport properties

### 4.1. Electrical contacts

**Figure 5.**
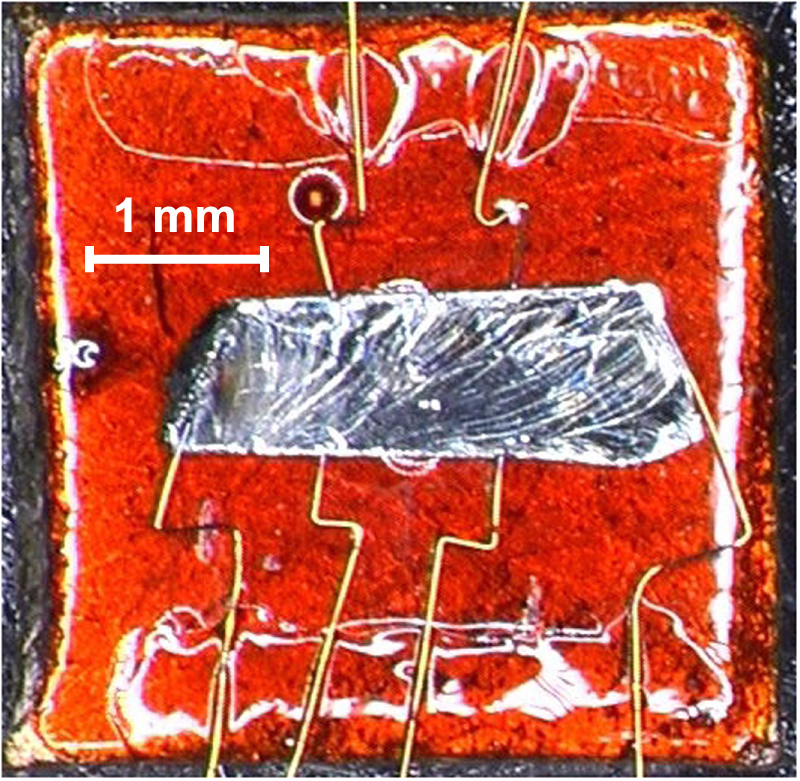
A sample with six welded contacts, which is mounted on a copper plate with insulation.

Measurements of transport properties are performed by the conventional six-probe method. Usually, for many chalcogenide topological insulators, electrical contacts are attached with silver paste such as 4922N by Dupont, which can be cured at room temperature. However, if Tl is included as a constituent element, then the contact resistance becomes higher than ∼15 Ω and it quickly degrades ∼1 k Ω after several hours. Indium solder can be used to make contacts with the contact resistance as low as ∼1 Ω, but the superconductivity of indium solder sometimes produces significant signals at low temperatures. To date, the spot-welding of gold wires is the best method for making electrical contacts, which become as low as ∼1 Ω and remain low for at least a few weeks. In the present experiment, 30-*μ*m-diameter gold wires were used as lead wires (figure 5), and the typical voltage for welding the wire was 5–9 V with a capacitor of 100 *μ*F.

### 4.2. Transport properties of TlBiSe_2_: optimization of synthesis conditions

**Figure 6.**
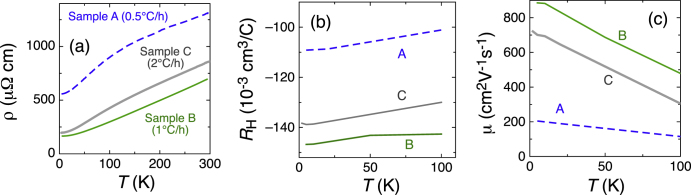
Transport properties of TlBiSe_2_ samples. Samples A, B, and C are grown at a temperature sweep rate of 0.5, 1, and 2 °C/h, respectively. Panels (a)–(c) present the temperature dependences of (a) 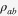, (b) *R*_*H*_, and (c) 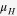.

Figures 6(a)–(c) show the temperature dependences of (a) resistivity, (b) the Hall coefficient, and (c) Hall mobility for three samples of TlBiSe_2_ single crystals grown in different conditions. Samples A, B, and C are grown at a temperature sweep rate of 0.5, 1, and 2 °C/h, respectively. The temperature dependences of the resistivity of all the three samples show a metallic behavior (figure 6(a)), and this indicates that the samples are degenerate semiconductors. Figure 6(b) shows that the temperature dependences of the Hall coefficient are not strong, and 4.2–6.2·10^19^ cm^−3^ 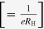 of *n*-type carriers are doped in this system (the Hall resistivity, 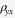, shows a linear *B*-dependence up to ±7 T). The growth-rate dependences of the transport properties are not simple; figure 6(a) shows that the resistivity of sample A grown by the slowest rate shows the highest resistivity of the three, but the fast-grown sample C does not show the lowest resistivity. The Hall coefficient also depends on the growth conditions, and there is a tendency for the absolute value of the Hall coefficient to decrease with increasing resistivity. This suggests that higher resistivity is not caused by a reduction in the charge carriers, but by an increase in disorder. Indeed, the sample A (B) of highest (lowest) resistivity shows the lowest (highest) Hall mobility (figure 6(c)). Apparently sample B is the best among the three samples, because it has lowest carrier concentration and disorder. On the other hand, I applied the growth conditions of sample C (the temperature sweep rate of 2 °C/h) to other TlBi(S
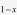
Se_*x*_)_2_ systems. The quality of sample C is certainly not the best among the three, but it is reasonably good because the mobility is more than three times of that of sample A. The data for lowest-mobility sample A are consistent with that by Mitsas *et al* [31], and thus in the present experiment the quality of samples is apparently improved. This is probably because of both the higher purity of the raw materials and the optimization of the growth conditions.

Now let us consider the origin of the growth-condition dependence of transport properties. Inductively-coupled plasma atomic-emission spectroscopy (ICP-AES) analysis is performed on samples A and B grown by the slow- and the middle-temperature sweep rates, respectively, to obtain the resultant chemical composition of crystals (table 1). The sum of the Tl and Bi contents is fixed to be 2. In both samples, bismuth composition is slightly larger than thallium, and this shows the substitution of 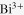 for 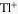. The fraction of the substitution is higher in sample A than in sample B, and thus it is naturally expected that this difference causes an increase in *n*-type carriers and imperfections by 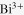 partially substituting 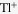 in sample A. In other words, it is important to suppress substitution of Bi for Tl to obtain better samples with less disorder.

**Table 1.** The data are obtained from ICP-AES analyses. The sum of thallium and bismuth contents is fixed to be 2.

TlBiSe_2_	Growth rate	Tl	Bi	Se
Sample	(°C/h)			
A	0.5	0.976 ± 0.008	1.024 ± 0.008	2.010 ± 0.003
B	1	0.985 ± 0.008	1.015 ± 0.008	2.002 ± 0.006

### 4.3. Transport properties of TlBiSe_2_

**Figure 7.**
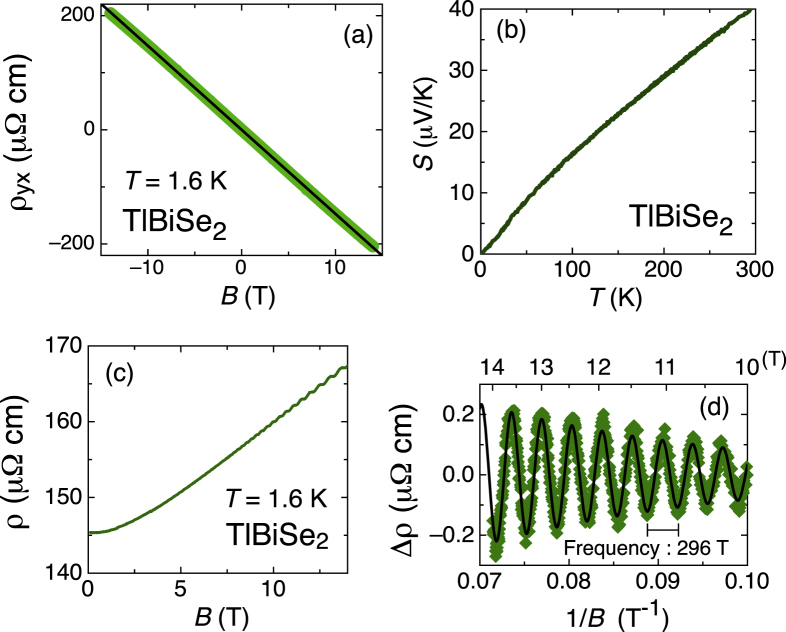
Transport properties of the best TlBiSe_2_ sample. (a) Field dependence of the Hall resistivity, 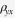. (b) Temperature dependence of the Seebeck coefficient. (c) Field dependence of the resistivity at 1.6 K. (d) Oscillatory component of magnetoresistance versus inverse of the magnetic fields. The solid line is a fit to data.

In this section, let me discuss the magneto transport of the best sample of TlBiSe_2_ (sample B in figure 6). Figure 7(a) shows the magnetic-field dependence of the Hall resistivity, 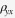, up to ±14 T. The solid line shows the linear fitting, and the lack of non-linearity (deviation from the linear fit is less than 1%) is indicative of the single-channel electronic transport. The carrier concentration of this sample is *n* = 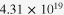 cm^−3^ [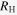 = −145 (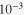 cm^3^/C)], and this result is in agreement with low-field data in figure 6(b). Please note that the bulk conduction channel must govern the transport properties of the present sample, and thus the topological surface channel does not play an important role, unfortunately. Figure 7(c) shows the field dependence of the magnetoresistance up to 14 T. The data are symmetrized with respect to the magnetic field. At very low fields, no anomaly is observed, and thus weak antilocalization behavior is missing in this system. This result is indicative of the dominance of the bulk-channel transport. At high fields, magnetoresistance shows a clear oscillation. Since the oscillatory component of the magnetoresistance shows a periodicity of 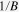, this behavior is Shubnikov-de-Haas (SdH) oscillation. The frequency of the SdH oscillation is *f* = 296 T, and this gives 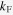 = 9.48 × 10^6^ cm^−1^ according to the Onsager relation, 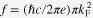. The 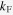 value is in good agreement with the ARPES result [21]. If a spherical Fermi surface (FS) is assumed, 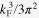 gives *n* = 2.88 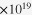 cm^−3^. This value is smaller than *n* obtained from the Hall coefficient, but it can be understood if the FS is elongated along the 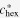-axis. Figure 3(b) shows the temperature dependence of the Seebeck coefficient, *S*. The negative sign of *S* is consistent with *n*-type doping in the present system.

### Transport properties of TlBi(S
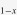
Se_*x*_)_2_

4.4.

**Figure 8.**
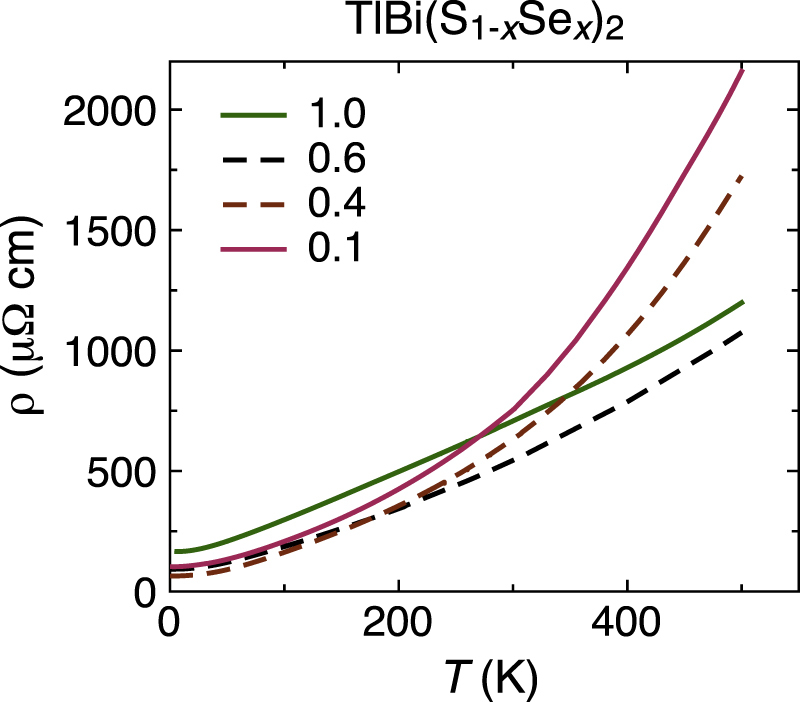
Temperature dependences of the resistivity for TlBi(S
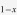
Se_*x*_)_2_, *x* = 1.0, 0.6, 0.4, and 0.1, up to 500 K.

From the transport properties at high temperatures, one may extract the energy gap if an activation behavior is observed [3]. In the present experiment I could not observe any activation behavior either in the resistivity or the Hall coefficient. However, the temperature dependence of resistivity shows some difference between Se-rich and Se-poor samples (figure 8). The resistivity above 300 K and the temperature dependences in *x* = 0.1 and 0.4 samples show a significant increase up to 500 K, whereas the increase is modest in *x* = 0.6 and 1.0 samples. In all these samples, the charge carriers are electrons, and thus the chemical potential is located in the conduction band. The observed behavior suggests that the responsible bands for the Se-rich composition and the other are different from each other, and thus it is suggested that band inversion occurs when *x* changes from 0.4 to 0.6 in the present system.

## 5. Summary

This article provides detailed information on the synthesis and characterization of TlBi(S
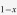
Se_*x*_)_2_. The growth condition of TlBiSe_2_ is optimized to obtain higher mobility using transport properties. XRD analysis confirmed that the change in the crystallographic structure is gradual in TlBi(S
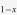
Se_*x*_)_2_, and this system is useful for studying the topological phase transition and related Dirac semimetals.

## Appendix A. Parameter and vector conversion of Tl-based ternary system and tetradymite topological insulators between hexagonal and rhombohedral unit-cell systems

The crystallographic structure of the Tl-based ternary system and tetradymite topological insulators can be expressed by either hexagonal or rhombohedral unit cells, which specify the structure by two independent lattice parameters. In the following, I show the conversion formulae from hexagonal to rhombohedral, and vice versa:

where  and  ( and ) are the lattice constants for hexagonal (rhombohedral) unit cells. In the hexagonal system, the *c*-axis is perpendicular to the layer-structure planes.

In the real space, the formulae for converting unit vectors between hexagonal and rhombohedral unit-cell systems are given as follows: Here, the unit vectors of a rhombohedral (hexagonal) system are expressed as , , and  (, , and ), and

is always valid. But,  and  depend on how to choose the rhombohedral unit-cell vectors. If the unit vectors of a rhombohedral system are taken as

then  is given by

The reversal conversion formulae are given by

and

By using these formulae, conversion of an axis is easily performed as follows.

The above conversion is in the real space, and here let us move to the reciprocal space. Here I show the conversion formulae for converting Miller indices as follows.

Note that the conversion formulae are quite different from those of the real space, because the direction of the reciprocal vector,  and , changes from that of the real space, unlike rectangular structures, such as cubic or orthorhombic. Those formulae naturally tell us the rule of absent reflection: if  (*n* is an integer), no reflection is observed at the position of the Miller index, because  does not become an integer.
